# Subjects with Impaired Fasting Glucose: Evolution in a Period of 6 Years

**DOI:** 10.1155/2014/710370

**Published:** 2014-08-20

**Authors:** E. Leiva, V. Mujica, R. Orrego, S. Wehinger, A. Soto, G. Icaza, M. Vásquez, L. Díaz, M. Andrews, M. Arredondo

**Affiliations:** ^1^Programa de Investigación de Excelencia Interdisciplinaria en Envejecimiento Saludable (PIEI-ES), Departamento de Bioquímica Clínica e Inmunohematología, Facultad de Ciencias de la Salud, Universidad de Talca, 3465548 Talca, Chile; ^2^Facultad de Medicina, Universidad Católica del Maule, Avenida San Miguel 3605, 3480112 Talca, Chile; ^3^Programa de Investigación de Excelencia Interdisciplinaria en Envejecimiento Saludable (PIEI-ES), Instituto de Matemáticas y Física, Universidad de Talca, Chile 2 Norte 685, 3465548 Talca, Chile; ^4^Laboratorio de Micronutrientes, INTA, Universidad de Chile, El Líbano 5524, Macul, 7830490 Santiago, Chile

## Abstract

*Aim*. To study the evolution of impaired fasting glucose (IFG), considering glucose and HbA1c levels and risk factors associated, in a period of 6 years. *Methods*. We studied 94 subjects with impaired fasting glucose (IFG) that were diagnosed in 2005 and followed up to 2012. Glucose and HbA1c levels were determined. A descriptive analysis of contingence charts was performed in order to study the evolution in the development of type-2 diabetes mellitus (T2DM). *Results*. Twenty-eight of ninety-four subjects became T2DM; 51/94 remained with IFG; and 20/94 presented normal fasting glucose. From the 28 diabetic subjects, 9 had already developed diabetes and were under treatment with oral hypoglycemic agents; 5 were diagnosed with plasma glucose < 126 mg/dL, but with HbA1c over 6.5%. In those who developed diabetes, 15/28 had a family history of T2DM in first relative degree. Also, diabetic subjects had a BMI significantly higher than nodiabetics (*t* test: *P* < 0.01). The individuals that in 2005 had the highest BMI are those who currently have diabetes. *Conclusion*. The IFG constitutes a condition of high risk of developing T2DM in a few years, especially over 110 mg/dL and in obesity patients.

## 1. Introduction

In Chile, according to the last National Health Survey, the prevalence of type 2 diabetes mellitus (T2DM) in Chilean adult population increased from 6.3% in 2003 up to 9.4% in 2009. The last survey also showed an increase of bad nourishing by excess from 63% to 67% in the same period. T2DM is one of the highest causes of morbidity, mortality, and damage of life quality, producing high economical costs [1]. T2DM prevalence has increased and every time is diagnosed in earlier ages, a tendency that has been continuous during the last years, although clinical studies have shown that it is possible to delay or prevent the development of T2DM by modifying the lifestyle and/or with pharmacological agents if they are applied on glucose intolerant population [2–4].

The increase of T2DM incidence in our country in a period of 6 years is high and worrying. Similar problems have been reported by Valdés et al. in Spanish population, where they also found a raise in the incidence of T2DM in the last years [5]. On the other hand, in studies carried out by our work group in 2005, a 6.0% (between 1007 randomly selected subjects) had diabetes and 26.3% of them presented alterations in fasting glucose [6].

For decades, the diagnosis of diabetes was based on plasma glucose criteria, fasting plasma glucose (FPG) or 2 h value in 75 g oral glucose test (OGTT) [7]. In 1997 and 2003, The Expert Committee on Diagnosis and Classification of Diabetes Mellitus [8, 9] recognized an intermediate group of individuals whose glucose levels are not fulfilling the criteria for diabetes diagnosis; however, their values are too high to be considered normal. These people were defined as having impaired fasting glucose (IFG) (100–125 mg/dL [5.6–6.9 mmol/L]) or impaired glucose tolerance (IGT) (2 h values in OGTT of 140–199 mg/dL [7.8–11.0 mmol/L]).

Individuals with IFG and/or IGT have been reported as having prediabetes, indicating the relatively high risk for the future development of T2DM. In 2003, the American Diabetes Association [10] proposes to reduce the high limit of normal glucose in plasma from 110 to 100 mg/dL. However, the World Health Organization did not accept the proposal and maintained their recommendation checked in 1999 with the level of 110 mg/dL as the normal maximum limit in fast [11]. The Latin-American Diabetes Association (ALAD) Clinical Guides [12, 13] as well as the Clinical Guide from Chilean Health Ministry suggested the diagnosis of IFG when the fasting plasma glucose is between 100 and 125 mg/dL [14]. Beyond the differences existing in order to establish the normal limit for fasting plasma glucose, the evidences coincide that glucose levels in the stage of prediabetic keep a direct relation with the risk of developing diabetes in the future, and this also constitutes an independent risk factor for cardiovascular diseases (CVD) [15, 16]. Both conditions, T2DM and CVD, are related with other factors such as dyslipidemia, arterial hypertension (HTA), abdominal obesity, sedentary, oxidative stress, metabolic syndrome (MS), and family data [17, 18].

Prediabetes condition includes IFG as well as IGT; both states have differences in their pathogenic origin, which also explains its epidemiological differences. The prevalence of both conditions varies significantly in different populations, from 6.3% in Chinese population [19] to 23.0% in Swedish population [20]; however, persistently, IGT occurs more frequently than IFG, and 30–60% of subjects with IGT have normal fasting glucose [21]. Furthermore, there is controversy about the progression of diabetes in IFG, ranging from 9.0 to 30.0% for periods from 5 to 10 years of evolution [22–25].

In 2009, a new recommendation was added in order to establish the diabetes and prediabetes diagnosis, accepting the measurement of glycated hemoglobin (HbA1c) certified by the National Glycohemoglobin Standardization Program (NGSP) and standardized or traceable to the Diabetes Control and Complications Trial (DCCT) reference assay, as a valid test to diagnose T2DM. The ADA 2010 [26], as well as WHO 2011 [27, 28], adopts the same recommendation and suggested that the levels of HbA1c ≥ to 6.5% may be considered as diagnoses of T2DM and levels lower than 5.6% are considered as normal. The intermediate values would correspond to states of high risk for developing diabetes (ADA, 2012) [7]. The Health Ministry of Chile, recently suggests not adopting this last one diagnosis criterion, as long as there is not a massive accreditation of the methodologies in the country for the performance of HbA1c [12, 13].

Our aim was to study the evolution of subjects with IFG that were studied by the “Programa de Investigación de Factores de Riesgo de Enfermedad Cardiovascular” (PIFRECV) [29], considering the plasma glucose and HbA1c levels and risk factors associated in a period of 6 years.

## 2. Methods

### 2.1. Subjects

The studied population was recruited in 2005 by the PIFRECV in 2005 [29]. For inclusion criteria in this study, those subjects that had a fasting glucose between 100 and 125 mg/dL in 2005 were contacted through phone calls, mail, and/or visits to their houses and invited to participate in an preliminary evaluation. The individuals that accepted to participate received information about the project and signed a written consent. This protocol was approved by the Ethical Committee of Universidad of Talca. Subjects that in 2005 had diabetes were excluded from the study, as well as individuals under corticoid treatment, pregnant women, and individuals with cardiovascular complications.

### 2.2. Clinical Data

In the present study a survey was performed that include (1) use of medications, specifically hypoglycemiants, antihypertensive, hypolipemiant, and corticoids; (2) family history of T2DM in first relative degree (sons, parents, or siblings); and (3) history of macrosomia and/or gestational diabetes (GD) in women.

### 2.3. Diagnostic Criteria

#### 2.3.1. Diabetes

Subjects were those with fasting glucose in plasma ≥126 mg/dL repeated in two consecutive days and/or HbA1c ≥ 6.5% and/or those persons that were under treatment with hypoglycemiants.

#### 2.3.2. IFG

Subjects with fasting glucose in plasma between ≥100 and <126 mg/dL. For the analysis, this group was subdivided in two groups: “low range” when fasting glucose in plasma was between 100 and 109 mg/dL and “high range” between 110 and 125 mg/dL.

#### 2.3.3. Risk Factors Related to Diabetes

Family history of T2DM in first relative degree, obesity or overweight, history of macrosomia, and/or gestational diabetes was considered.

### 2.4. Laboratory Measures

Fasting glucose levels in plasma (enzymatic Kit Gluco-quant; glucose/HK Roche, Mannheim, Germany) and glycosylated hemoglobin A1c (HbA1c) levels in total blood (immune turbidimetric method according to HbA1C III, Tina-quant. Hemoglobin A1C III, standardized according to IFCC, Roche, Mannheim, Germany) were processed in a Hitachi 902 Automatic Analyzer (Roche Diagnostics Mannheim, Germany).

### 2.5. Statistical Analysis

A descriptive and relational analytic analysis was made, through the analysis of contingence charts, in order to study the effect of different factors in the development of T2DM. *t*-test was used for two group analyses; for contingency tables we used Fisher's exact test (2 × 2) or chi-square (4 × 2). Cox analysis for hazard ratio (HR) was performed for glycemia, triglycerides, LDL-cholesterol, and BMI and adjusted for tabaquism, familiar history, and medications intake. A significant difference was considered when *P* was <0.05. The data were analyzed with the software SPSS version 14.0.

## 3. Results and Discussion

### 3.1. Results

#### 3.1.1. Patient Characteristics

Of the 177 patients who fulfilled the inclusion criteria, 7 were dead, 38 were impossible to contact due to a change of address, and 38 rejected their participation. Ninety-four subjects (54 female and 40 male) with IFG diagnosed in 2005 accepted to participate in this study. The participants had a range of age between 25 and 80 years old and there were no differences between those whom became T2DM and not T2DM. Twenty-eight of ninety-four subjects became type 2 diabetic patients, 43 persisted with IFG, and 23 presented normal fasting glucose. From the 28 diabetic subjects, 19 were diagnosed by the present study whereas 9 had already developed diabetes and were under treatment with oral hypoglycemic (glybenclamide and/or metformin).

When the individuals were grouping according to their fasting glucose levels from 2005, we found that 66/94 were in a low range (<110 mg/dL) and 28/94 in high range (>110 mg/dL) of glycemia. From the low range group, 17/66 developed T2DM in 2011, while 22/66 got normal; in the high range group, 11/28 developed T2DM and nobody got normal, test *P* < 0.001 (Table 1); these data include 9 subjects with T2DM at the time of the study were treated with oral hypoglycemic drugs, for this reason some of them showed glycemia below 126 mg/dL.

In relationship to the criteria for diabetes diagnosis in those newly diagnosed and untreated (*n* = 19), nine share both criteria for diagnose (glucose levels and HbA1-c altered), five have only glucose levels > 126 mg/dL, and five have only HbA1c > 6.5%. The mean of HbA1c in diabetic patients was significantly higher than nondiabetics patients (7.3% versus 5.4%) (*t*-test *P* < 0.01).

#### 3.1.2. Family History

Respect to the family history, in those who developed diabetes 15/28 had a family background of T2DM in first relative degree, versus 13/28 in those non-diabetics (*P*: NS) (Table 2). The age of the subjects in both groups was no different (*t*-test *P* = 0.507). Regarding obstetric history, no conclusions were obtained from the information about macrosomia and/or gestational diabetes due to the high level of ignorance of the topic by the subjects.

#### 3.1.3. Anthropometry Data and Blood Pressure

BMI in diabetic patients was significantly higher than nondiabetic patients (*t*-test *P* < 0.01) and those subjects that in 2005 had the highest BMI are the same that currently have diabetes. The circumference waist in diabetic patient was significantly higher than nondiabetics (*t*-test *P* < 0.05), but in 2005 there was no difference between the same groups. Also, there were no differences in blood pressure in 2011 between diabetic and nondiabetic patients (Table 3); however, in 2005 diastolic pressure was significantly higher in nondiabetics.

#### 3.1.4. Risk Factor for Type 2 Diabetes

The risk factor for type 2 diabetes was increased in subjects that in 2005 had a high glycemia (HR: 2.06; CI: 1.76–5.14; *P* < 0.03 (Table 4)). LDL cholesterol, triglycerides, and BMI were not significant.

### 3.2. Discussion

IFG is an intermediate state of control of glycemia which implies a high risk of developing diabetes in the following years. We proposed to study the evolution after 6 years of subjects with IFG [29]. Our results showed that 28/94 of the subjects with IFG in 2005 now have diabetes. Nearly half of them (43/94) remained as IFG and 23/94 got normal despite not receiving any interventions. A prevalence of 30% is much higher than the general population, suggesting that having IFG is a major risk factor for developing T2DM. From the classic factors related to diabetes development, the presence of obesity and high waist circumference were not relevant factors in this population; despite that, waist circumference was high in all patients at 2005 and increased in 2011.

In diabetic group, we observed a tendency to present higher frequency of family history in first or second relative degree. In this group, all of them had altered glucose levels and therefore a high predisposition was expectable, because they have a genetic charge expressed in their family diabetes history.

Interestingly, we observed that, from 28 subjects who had levels of glycemia between 110 to 125 mg/dL, twelve (39.3%) now are diabetics, whereas only sixteen (25%) that were classified on the lower range of hyperglycemia are diabetics at present. In this last group, more than a third improved their status, while in the higher range of glycemia none of the subjects improved. These results are similar to the Hoorn Study [22] who monitoring for 5.8–6.5 years and found a 33.0% of isolated IFG progression (110 to 125 mg/dL) to diabetes. Eschwège et al., in a monitoring of 30 months, reported a 14.9% of isolated IFG progression to DM [23], and Vaccaro et al. reported lower values of progression (9.1%) to diabetes in 11.5 years of monitoring [24]. Baena-Diez et al. in a cohort study with a monitoring of 10 years showed that half of the cases with IFG normalized their glucose levels, whereas 28.7% developed diabetes [25].

All the studies mentioned were performed considering IFG with values of 110 to 125 mg/dL. Nichols et al. compared the progression to diabetes from IFG in a cohort that was followed for 6.3 years and classified according to the initial glucose levels, finding an 8.1% of progression to diabetes with IFG of 100 to 109 mg/dL and 24.3% of progression with IFG of 110 to 125 mg/dL [30]. Our study was different in number of patients studied and design, because the patients were studied in a transverse cut where different alterations were diagnosed in the basal glucose levels, without knowing the time of previous evolution. Our results report the progression from IFG to diabetes in a group of adults in the last 6 years, giving additional information, especially for our country and Latin-American countries that can be useful as a complement for better assessing the diabetes risk according to the level of hyperglycemia and the nutritional state.

On the other hand, we know that the measuring of HbA1c recently has been accepted for diabetes diagnosis, and we observed that both criteria-glucose levels and HbA1c were consistent for the 50.0% of the cases, whereas around 25.0% of the cases had only one of the criteria altered; then without the use of the HbA1c we would have found less than 25.0% of diabetes in our group.

## 4. Conclusions

In summary, we propose that IFG constitutes a condition of high predisposition in order to develop T2DM, especially in subjects with glycemia over 110 mg/dL and with central and/or general obesity. However, to establish individual predisposition it is necessary to consider the analysis of a set of parameters for each person.

## Figures and Tables

**Table 1:** Relation according to the range of fasting glucose levels (FGL).

	2011FGL (mg/dL)				Total
	<100	100–109	110–125	≥126	
2005FGL mg/dL					
100–109 *N* (%)	21 (31.8)	16 (24.2)	17 (25.8)	12 (18.2)	66 (100)
110–125 *N* (%)	0 (0.0)	5 (17.9)	16 (57.1)	7 (25.0)	28 (100)

Total	21 (22.3)	21 (22.3)	33 (35.1)	19 (20.2)	94 (100)

This table includes T2DM subjects under treatment with hypoglycemic drugs (*n* = 9).

∗Chi-square test: *P* value < 0.001.

**Table 2:** Comparison between diabetics and no diabetics according to gender and family history of T2DM in first relative degree.

	Diabetics		Nondiabetics		*P* value
	*N*	%	*N*	%	
Gender					
Female	19	67.8	35	53.0	NS
Male	9	32.2	31	47.0	
Total	**28**	**100**	**66**	**100**	
Family history of T2DM					
A	15	53.5	27	40.9	NS
B	13	46.5	39	59.1	
Total	**28**	**100**	**66**	**100**	

Fisher's test: NS: not significant; A: with record; B: without record.

**Table 3:** Comparison between diabetics and nondiabetics according to clinical antecedents.

	Year	Diabetics		Nondiabetics		*P* value^1^
		$\overset{-}{x}$	*S*	$\overset{-}{x}$	*S*	
Age		59.07	10.47	60.79	11.81	NS
Systolic pressure (mmHg)	2005	133.64	16.18	142.48	21.51	0.054
Systolic pressure (mmHg)	2011	145.00	19.61	149.48	20.25	NS
Diastolic pressure (mmHg)	2005	76.61	10.10	84.36	12.28	<0.01
Diastolic pressure (mmHg)	2011	82.43	11.02	82.13	9.55	NS
BMI (kg/m^2^)	2005	33.10	4.27	30.40	4.36	<0.01
BMI (kg/m^2^)	2011	33.28	4.00	30.39	4.24	<0.01
Waist (cm)	2005	96.50	8.65	99.19	11.42	NS
Waist (cm)	2011	109.23	8.63	103.45	10.70	<0.05

^1^ *t* test: NS: not significant.

**Table 4:** Cox multivariate analysis for type 2 diabetes mellitus risk factor.

Variable	Hazard ratio	IC	*P* value
BMI 2005	1.87	0.84–3.18	0.08
Glycemia 2005	2.06	1.76–5.14	0.03
TGC 2005	1.03	0.93–3.91	0.25
LDL chol 2005	1.11	0.97–4.64	0.10

BMI: body mass index; TGC: triglycerides; LDL chol: LDL cholesterol

adjusted by tabaquism, use of medications, and family history.
